# Serum Complement C1q Activity Is Associated With Obstructive Coronary Artery Disease

**DOI:** 10.3389/fcvm.2021.618173

**Published:** 2021-04-29

**Authors:** Shuren Guo, Xiaohuan Mao, Xiaohua Li, Huan Ouyang, Yuhua Gao, Liang Ming

**Affiliations:** ^1^Department of Clinical Laboratory, The First Affiliated Hospital of Zhengzhou University, Zhengzhou, Henan, China; ^2^Department of Clinical Laboratory, Henan Provincial People's Hospital, People's Hospital of Zhengzhou University, Zhengzhou, Henan, China; ^3^Department of Clinical Laboratory, ShenQiu People's Hospital, ShenQiu, Henan, China

**Keywords:** serum complement C1q, obstructive CAD, Gensini score, restenosis, non-obstructive CAD

## Abstract

**Background:** Complement C1q plays a dual role in the atherosclerosis. Previous studies showed inconsistent results about the association of serum C1q levels and coronary artery disease (CAD). Here, we explored the associations of serum C1q activity with CAD, coronary stenosis severity, cardiovascular biomarkers, and 1-year restenosis after coronary artery revascularization.

**Methods:** We enrolled 956 CAD patients and 677 controls to evaluate the associations of serum complement C1q activity to the presence and severity of obstructive CAD and non-obstructive CAD. Serum C1q activity and the concentrations of laboratory markers were measured in all subjects. All the data were analyzed using SPSS22.0 software.

**Results:** Serum C1q activity in Obstructive CAD and Non-Obstructive CAD groups was significantly higher than the control group (195.52 ± 48.31 kU/L and 195.42 ± 51.25 kU/L vs. 183.44 ± 31.75 kU/L, *P* < 0.05). Greater C1q activity was significantly correlated with higher total cholesterol (TC) and triglyceride (TG) levels. C1q activity was associated with an increased Odds Ratio (OR) of CAD (OR = 1.322, 95% CI 1.168–1.496, *P* < 0.05) and 1-year restenosis after revascularization (the highest OR = 3.544, 95% CI 1.089–12.702, *P* < 0.05). Complement C1q activity was not correlated with Gensini score in the Obstructive CAD group after adjustment for confounders. C1q activity has low value in predicting the incidence of CAD.

**Conclusion:** Serum complement C1q activity is associated with obstructive CAD.

## Introduction

Coronary artery disease (CAD) is projected as one of the leading causes of disease burden in the world in 2030 (1). Atherosclerosis (AS) is a pathological change throughout the whole pathogenesis of CAD. The formation of atherosclerotic plaque is related to endothelial dysfunction, large amount of lipid deposition in the arterial wall, aggravation of immune response, proliferation of vascular smooth muscle cells, and accumulation of extracellular matrix. Studies demonstrated that the complement system (CS) triggered inflammatory response and participates in the occurrence and development of AS (2). Atherosclerotic lesions contained several triggers such as C-reactive protein for the activation of the complement cascade. Previous studies indicated that the classical pathway rather than the alternative pathway was activated in plaques (3).

Complement C1q is a complex glycoprotein composed of 18 polypeptide chains. The C-terminal globular head region mediates the recognition of different molecular structures, and the N-terminal collagen like tail mediates the immune effector mechanism. As one of the starting components of classical complement pathway, C1q plays positive and negative effects on AS (4). Many experimental studies have shown that C1q played a beneficial role in the early stage of AS. C1q binds apoptotic cells and cell debris from atherosclerotic plaques, and plays an important role in their disposal (5–9). C1q played a beneficial role in early atherosclerosis by regulating macrophage molecular signal through complement independent pathway and modulating the uptake of atherogenic lipoprotein. On the contrary, C1q might be involved in accelerating the formation of atherosclerosis due to its proinflammatory effect (4). C1q could have a role in regulating collagen-induced platelet activation, production of reactive oxygen species (ROS), and associated leukocyte recruitment during vessel wall injury (10). Previous studies showed inconsistent results on the association of serum complement C1q level and the severity of different types of CAD with variant study groups and small sample size (11–13). In this study, we enrolled a total of 956 CAD patients and 677 healthy controls to assess the associations of serum C1q activity with the stenosis degree of coronary artery, cardiometabolic phenotypes, and the incidence of CAD and 1-year restenosis.

## Subjects and Methods

### Subjects

We recruited 1,336 patients who received coronary angiography (CAG) from June 2019 to June 2020 in The First Affiliated Hospital of Zhengzhou University at Henan Province. According to arteriography, patients were divided into normal or near-normal coronary arteries (NNCAs, *n* = 380), non-obstructive CAD group (*n* = 153), and obstructive CAD group (*n* = 803). NNCAs were defined as having all coronary artery stenosis ≤20%. According to previous studies, non-obstructive CAD was defined as having at least one coronary artery stenosis between 21 and 49% (14–16). Obstructive CAD was defined as angiographically demonstrated stenosis of >50% in major coronary vessel, with or without clinical symptoms of angina, negative changes of cardiac biomarkers, and typical electrocardiographic patterns (17). The severity of coronary artery stenosis was quantified by the number of diseased vessels with ≥50% stenosis and modified Gensini scores. We first categorized each patient by the number of diseased vessels with ≥50% stenosis in a single, double, or triple-vessel distribution. We defined vessel distribution by the left anterior descending artery and its branches, the left circumflex artery and its branches, and the right coronary artery and its branches. Patients with ≥50% stenosis of left main coronary artery were classified into the three-vessel obstructive CAD group (18). So, we finally created four categories of CAD extent: non-obstructive CAD, 1-, 2-, 3-vessel/left main obstructive CAD.

The control group consisted of 677 healthy persons without cardiovascular disease via physical examination including cardiac echocardiography, chest CT, and electrocardiogram every 2 years. The controls were frequency-matched to the cases on age and sex. Subjects were excluded if they (1) underwent percutaneous coronary intervention, coronary bypass surgery, or angioplasty; (2) had cardiac diseases like cardiomyopathy, valvular or congenital heart disease, heart failure, coronary spasm, or myocardial bridge; (3) patients who had malignant tumors, acute or chronic infection, autoimmune disease; (4) patients whose blood pressure ≥180/110 mmHg after taking standard antihypertensive drugs, patients whose ALT or AST was three times higher than normal, patients whose estimated glomerular filtration rate (eGFR) <15 mL/(min·1.73 m^2^). The study protocol followed the declaration of Helsinki, and The Zhengzhou University Committee approved this study (approval number 2020-KY-172).

During the study period, 485 of 956 patients underwent percutaneous coronary intervention (PCI) due to the presence of indications for revascularization. Re-stenosis was defined as having a >50% decrease in the luminal diameter as determined by quantitative coronary angiography. These 485 patients were followed up for 12 months after revascularization to track restenosis ratios. The follow-up was ascertained through telephone interviews with participants and verification by checking medical records at 30 days and 6 months after revascularization.

### Collection and Definition of Clinical Variables

Demographic factors including age, sex, smoking status (ever and never), alcohol drinking status (ever and never), type of diabetes management (lifestyle modification, oral agents, insulin, and oral agents plus insulin), and medicines were documented by checking participants' medical records and questionnaires. Weight and height were measured on the day of enrollments. Dyslipidemia and hypertension were defined based on the previously published guidelines (19, 20). Venous blood samples were collected from upper limbs before CAG. Serum complement C1q activity was tested by immunity transmission turbidimetry on a Cobas 8000 Analyzer (Roche Diagnostics, Germany). Fasting plasma glucose (FPG), low-density lipoprotein cholesterol (LDL-C), high-density lipoprotein cholesterol (HDL-C), total cholesterol (TC), triglycerides (TG), high sensitive C-reactive protein (hs-CRP), liver function, and renal function were measured on a Cobas 8000 Analyzer (Roche Diagnostics, Germany) using standard methods. Hemoglobin A1c (HbA1c) was assayed by high-performance liquid chromatography on a Premer-Premier Hemoglobin Testing System.

Selective CAG was performed in all patients using the standard Judkins technique. The localization of coronary artery disease and the rate of lumen stenosis were determined by CAG. Gensini score of coronary artery equals the sum of all segment scores. Each segment score equals segment weighting factor multiplied by a severity score. Segment weighting factor assigned to specific coronary segment are five for left main coronary artery, 2.5 for proximal left anterior descending coronary artery (LAD) and proximal left circumflex branch, 1.5 for mid-segment of LAD, 0.5 for second diagonal branch and posterolateral branch, and one for other branches. Severity score allocated to the definite percentage luminal diameter reduction are 1 for 0–25%, 2 for 26–50%, 4 for 51–75%, 8 for 76–90%, 16 for 91–99%, and 32 for 100% stenosis (21). We used the Gensini score as a dependent variable to describe and judge the severity of coronary stenosis.

### Statistical Analysis

Continuous variables were expressed as mean ± standard deviation (normally distributed data), median and quartile spacing [M (P25-P75)] (non-normally distributed data). Categorical variables are expressed as the frequency and its percentage. Continuous variables were analyzed using Student's *t*-test in normally distributed data, and Mann-Whitney test in non-normally distributed data. Categorical variables were analyzed using the Chi-square test. All the statistical analyses were executed using Statistical Package for Social Science (SPSS, version 22.0). The association of C1q with obstructive CAD and 1-year restenosis was analyzed by logistic regression models with three progressive degrees of adjustment. Model 1 was a crude model without any confounders; model 2 was adjusted for age, sex, and cardiovascular risk factors including smoking habit, alcohol drinking habit, overweight, hypertension, and dyslipidemia; model 3 was additionally adjusted for laboratory tests including HbA1c, FPG, alanine aminotransferase (ALT), aspartate aminotransferase (AST), urea nitrogen (UREA), uric acid (UA), estimated glomerular filtration rate (eGFR), homocysteine (HCY), and lipid profiles. For each model, C1q was first analyzed as a continuous variable, and then as an ordinal variable based on its quartile distribution. A two-sided value of *P* < 0.05 was considered statistically significant.

## Results

### Clinical Characteristics

**Figure 1:**
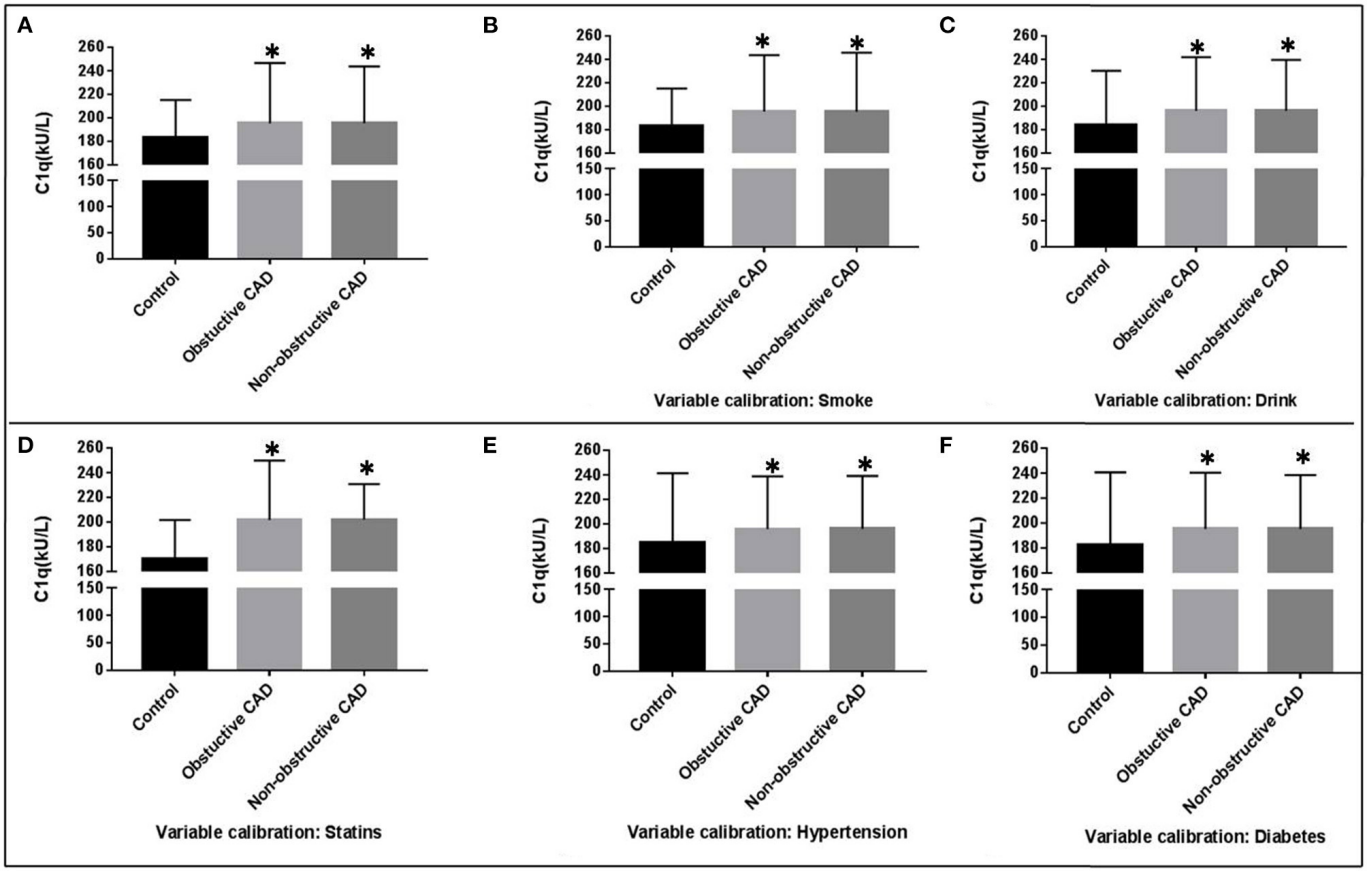
Covariance analysis of C1q activity among the three groups. *P < 0.05.

The general characteristics of the study groups were detailed in Table 1 and Supplementary Table 1. There was no statistically significant difference between CAD group and controls for age and gender. Overall, CAD patients were older, more likely to be smokers, obese, hypertensive, dyslipidemia, and hyperglycemia. Comparing with the controls, individuals in the CAD group showed significantly lower plasma concentrations of Total proteins (TP), Albumin (ALB), TC, HDL-C, and ApoA. Diastolic blood pressure (DBP), systolic blood pressure (SBP), body mass index (BMI), and the plasma concentrations of HbA1c, FBP, TG, C1q, liver function, and renal function were significantly higher in the CAD group compared to the control (*P* < 0.05). Complement C1q activity in obstructive CAD and non-Obstructive CAD groups identified as 195.52 ± 48.31 kU/L and 195.42 ± 51.25 kU/L, were significantly higher than the control group (183.44 ± 31.75 kU/L, *P* < 0.05) (Table 1, Figure 1). Even after adjusting for after calibrating smoke, drink, hypertension, dyslipidemia, diabetes, and the history of taking statins, serum complement C1q activity in the CAD groups were significantly higher than in the control group (*P* < 0.05; Figure 1). However, there was no significant difference in the four categories of CAD extent for C1q, Pro-Brain Natriuretic Peptide (Pro-BNP), and high sensitive cardiac troponin T (TNT-HS) (Table 1). There was a significant higher proportion of documented stable angina in the non-obstructive CAD than that in the obstructive CAD group (59.5 vs. 49.5%).

**Table 1:** Clinical characteristics of patients.

			**Number of stenosis**		
	**Non-obstructive CAD (*n* = 153)**	**Obstructive CAD (*n* = 803)**	**1 (*n* = 250)**	**2 (*n* = 189)**	**3/LMCD** **(*n* = 364)**
Smoking	43 (28.1)	279 (34.7)	93 (37.2)	65 (34.4)	121 (33.2)
Drinking *n* (%)	46 (30.1)	242 (30.1)	84 (33.6)	57 (30.2)	101 (27.7)
Hypertension *n* (%)	57 (37.3)	404 (50.3)	129 (51.6)	93 (49.2)	182 (50)
Clopidogrel *n* (%)	0 (0)	9 (1.1)	2 (0.8)	1 (0.5)	9 (2.5)
ACE inhibitors *n* (%)	0 (0)	12 (1.5)	2 (0.8)	3 (1.6)	7 (1.9)
ARB *n* (%)	17 (11.1)	129 (16.1)	31 (12.4)	36 (19)	62 (17)
β-blockers *n* (%)	14 (9.2)	64 (8)	17 (6.8)	15 (7.9)	32 (8.8)
Calcium channel blockers *n* (%)	12 (7.8)	81 (10.1)	18 (7.2)	15 (7.9)	48 (13.2)
Diuretics *n* (%)	4 (2.6)	31 (3.9)	6 (2.4)	7 (3.7)	18 (4.9)
Diabetes *n* (%)	44 (28.8)	276 (34.4)	82 (32.8)	66 (34.9)	128 (35.2)
Diabetes management					
Lifestyle modification *n* (%)	15 (9.8)	83 (10.3)	3 (1.2)	8 (4.2)	12 (3.3)
Oral agents only *n* (%)	14 (9.2)	61 (7.6)	13 (5.2)	10 (5.3)	38 (10.4)
Insulin only *n* (%)	13 (8.5)	119 (14.8)	32 (12.8)	30 (15.9)	57 (15.7)
Oral agents and insulin *n* (%)	2 (1.3)	13 (1.6)	2 (0.8)	1 (0.5)	11 (3)
Family History of CAD *n* (%)	30 (19.6)	160 (18.9)	30 (12)	40 (21.2)	90 (24.7)
Dislipidemia *n* (%)	30 (19.6)	152 (19.9)	54 (21.6)	33 (17.5)	65 (17.9)
Statins *n* (%)	19 (12.4)	126 (15.7)	30 (12)	25 (13.2)	51 (14)
Rehospitalized ratio	10 (6.5)	181 (22.5)	37 (14.8)	49 (25.9)	95 (26.1)
Stable angina	91 (59.5)^*^	391 (49.5)^*^	122 (48.8)	89 (47.1)	180 (49.5)
Acute coronary syndrome	62 (40.5)^*^	412 (50.5)^*^	128 (51.2)	100 (52.9)	184 (50.5)
Unstable angina pectoris	40 (26.1)	279 (34.3)	78 (31.2)	76 (40.2)	125 (34.3)
Myocardial infarction on presentation	22 (14.4)	133 (16.6)	50 (20)	24 (12.7)	59 (16.2)
ST-segment elevation myocardial infarction	15 (9.8)	71 (9.3)	26 (10.4)	11 (5.8)	34 (9.3)
Non-ST-segment elevation myocardial infarction	7 (4.6)	62 (6.9)	24 (9.6)	13 (6.9)	25 (6.9)
BMI(kg/m^2^)	23.86 ± 2.4	23.96 ± 2.33	23.72 ± 2.26	24.01 ± 2.3	24.1 ± 2.4
SBP(mmHg)	129.79 ± 19.71	128.82 ± 17.38	128.13 ± 18.68	126.17 ± 15.83^*^	130.71 ± 17.1^#^
DBP(mmHg)	78.54 ± 13.28	77.72 ± 10.93	77.05 ± 11.77	76.34 ± 10.37	78.9 ± 10.55
HR(heart ratio)	75.44 ± 10.75	75.12 ± 10.19	73.73 ± 9.73	75.29 ± 10.9	76.04 ± 10.02
Gensini Score	16.13 ± 5.69	38.77 ± 31.32	25.85 ± 17.55^#^	39.23 ± 18.01^*^	63.85 ± 28.57^§^
HbA1C(%)	5.98 ± 1.12^*^	6.19 ± 1.31	5.94 ± 1.07^*^	6.28 ± 1.37^#^	6.28 ± 1.38^#^
FPG (mmol/L)	5.44 ± 1.76	5.57 ± 1.89	5.43 ± 1.69	5.44 ± 1.62	5.71 ± 2.11
ALT (U/L)	30.25 ± 33.92	43.25 ± 189.45	35.19 ± 40.17	48.47 ± 210.24	44.5 ± 215.19
AST (U/L)	29.13 ± 27.18	44.62 ± 302.62	30.48 ± 27.42	30.06 ± 49.25	55.32 ± 398.44
TBIL (μmol/L)	14.4 ± 22.31^*^	10.81 ± 7.12^#^	10.71 ± 5.85^#^	12.34 ± 11.49	10.3 ± 5.21^#^
BILD (μmol/L)	7.11 ± 18.39^*^	4.78 ± 3.58	4.85 ± 3.05	5.48 ± 6.31	4.5 ± 2.09^#^
GGT (U/L)	41.17 ± 53.48	33.61 ± 34.15	34.72 ± 42.82	33.07 ± 37.09	33.39 ± 29.07
TP (g/L)	66.94 ± 6.29^*^	65.64 ± 5.37	65.45 ± 5.53^#^	65.71 ± 4.56	65.69 ± 5.59^#^
ALB (g/L)	41.42 ± 4.48	40.66 ± 4.14	40.61 ± 4.16	40.74 ± 3.61	40.66 ± 4.33
ALP (U/L)	77.64 ± 51.32	75.6 ± 24.88	73.09 ± 20.08	73.13 ± 19.76	77.45 ± 27.91
TC (mmol/L)	4.04 ± 1.03^*^	3.81 ± 1.04	4 ± 1	3.72 ± 1.04^#^	3.72 ± 1.05^#^
TG (mmol/L)	1.84 ± 1.65	1.6 ± 1.14	1.55 ± 0.86	1.63 ± 1.33	1.61 ± 1.2
HDL (mmol/L)	1.1 ± 0.34^*^	1.03 ± 0.29	1.07 ± 0.31	1.02 ± 0.29^#^	1.01 ± 0.26^#^
LDL (mmol/L)	2.33 ± 0.93^*^	2.14 ± 0.85	2.27 ± 0.79^*^	2.07 ± 0.89^#^	2.1 ± 0.86^#^
APOA (g/L)	1.21 ± 0.46^*^	1.15 ± 0.26	1.2 ± 0.28^*^	1.15 ± 0.24	1.12 ± 0.24^#^
APOB (g/L)	0.92 ± 0.52	0.93 ± 1.92	0.82 ± 0.26	1.23 ± 4.11	0.86 ± 0.33
Lpa (g/L)	0.22 ± 0.25^*^	0.31 ± 1.11	0.3 ± 0.31	0.52 ± 2.35^#^	0.22 ± 0.23^*^
HCY (umol/L)	14.5 ± 6.8	14.98 ± 8.68	14.96 ± 10.44	13.95 ± 6.67	15.53 ± 8.48
hsCRP (mg/L)	3.91 ± 9.14	7.55 ± 21.16	2.37 ± 5.24	6.46 ± 19.47	10.14 ± 25.23
Creatinine (μmol/L)	72.26 ± 23.9	80.7 ± 67.75	77.87 ± 54.87	82.11 ± 67.9	81.25 ± 72.31
Urea mmol/L)	5.85 ± 3.01	5.83 ± 2.96	5.97 ± 2.66	5.59 ± 2.81	5.87 ± 3.13
Uric acid (mmol/L)	309.47 ± 99.24	304.17 ± 105.06	297.33 ± 103.91	310.8 ± 98.85	304.27 ± 108.03
eGFR	91.06 ± 19.02	88.7 ± 19.72	91.07 ± 18.02	89 ± 20.5	87.66 ± 20.04
C1q (kU/L)	195.42 ± 51.25	195.52 ± 48.31	177.13 ± 41.98	189.13 ± 41.67	195.9 ± 45.93
Pro-BNP (ng/L)	1019.37 ± 3846.95	978.33 ± 2200.01	1744.79 ± 3587.73	1001.98 ± 1630.1	815.19 ± 1187.18
TNT-HS (ng/L)	1.14 ± 18.8	1.26 ± 13.33	1.07 ± 2.13	2.89 ± 25.52	1.33 ± 1.48

*SBP, systolic blood pressure; DBP, diastolic blood pressure; BMI, body mass index; ALT, alanine aminotransferase; AST, aspartate aminotransferase; TC, total cholesterol; TG, triglyceride; HDL-c, high density lipoprotein-c; LDL-c, low density lipoprotein-c; ApoA, Apolipoprotein A; ApoB, Apolipoprotein B; HCY, homocysteine; FBG, fasting blood glucose; ACE, angiotensin converting enzyme; ARB, angiotensin receptor blocker. FPG, fasting plasma glucose; HbA1c, glycosylated hemoglobin; hsCRP, high sensitive C-reactive protein; Pro-BNP, Pro-Brain Natriuretic Peptide; TNT-HS, high sensitive cardiac troponin T*.

*P < 0.05 vs. #.

### C1q and Cardiometabolic Phenotypes

We tested 14 biomarkers, i.e., blood lipids (LDL-C, HDL-C, TC, and TG), blood glucose (FPG, HbA1c), blood pressure (systolic and diastolic), proinflammatory measures (hs-CRP), and adiposity measure (BMI). Overall, greater C1q activity was significantly correlated with higher TC levels (β = 0.001, *P* = 0.009) and TG (β = 0.002, *P* = 0.031), but not with 12 other biomarkers after adjustment for confounders (Table 2).

**Table 2:** Associations of C1q with 14 cardiovascular biomarkers in CAD patients.

**Variables**	***n***	**β (95% CI)**	***P***
HCY (umol/L)	956	0.007 (−0.01–0.024)	0.401
Blood lipids			
T-CHO (mmol/L)	956	0.001 (−0.001–0.003)	**0.009**
TG (mmol/L)	956	0.002 (0–0.004)	**0.031**
HDL (mmol/L)	956	0 (0–0.001)	0.805
LDL (mmol/L)	956	0.001 (0–0.002)	0.181
APOA (g/L)	956	0 (0–0.001)	0.704
APOB (g/L)	956	0 (−0.001–0.001)	0.857
Lpa (g/L)	956	0 (−0.003–0.003)	0.863
Blood glucose			
HbA1C (%)	956	0.000 (−0.002–0.002)	0.721
FPG (mmol/L)	956	−0.001 (−0.004–0.001)	0.316
Blood pressure			
SBP (mmHg)	956	−0.01 (−0.037–0.017)	0.466
DBP (mmHg)	956	−0.005 (−0.023–0.012)	0.55
Inflammation			
hsCRP (mg/L)	956	0.018 (−0.036–0.073)	0.51
Adiposity			
BMI (m/kg^2^)	956	0.003 (−0.001–0.007)	0.139

*Multivariable linear regression was performed with the C1q as the independent variable and each cardiovascular biomarker as the dependent variable, with adjustment for age, sex, smoking habit, alcohol drinking habit, hypertension, diabetes status, dyslipidemia, medicine, and family history of CAD*.

*The bold values indicate significant results with P < 0.05*.

### C1q Activity and Coronary Stenosis Severity in Obstructive CAD as Determined by Gensini Scores

We analyzed the correlation between the serum complement C1q activity and Gensini score. Spearman analysis showed no significant direct correlation (*P* > 0.05) in the non-obstructive CAD. The regression model of complement C1q was established in the obstructive CAD samples (Table 3).

**Table 3:** Linear regression analysis of Gensini score.

**Models**	**Factors**		**β (95% CI)**	***P***
Model 1	C1q in obstructive CAD		0.083 (0.035–0.131)	<0.001
	C1q in CAD subgroup according to the number of stenos			
		1	−0.003 (−0.079–0.074)	0.942
		2	0.228 (0.072–0.385)	**0.006**
		3	0.08 (−0.019–0.18)	0.111
Model 2	C1q in obstructive CAD		0.048 (0.011–0.085)	**0.012**
	C1q in CAD subgroup according to the number of stenos			
		1	−0.016 (−0.143–0.112)	0.8
		2	0.18 (−0.273–0.633)	0.409
		3	0.043 (−0.073–0.159)	0.465
Model 3	C1q in obstructive CAD		0.025 (−0.026–0.076)	0.333
	C1q in CAD subgroup according to the number of stenos			
		1	−0.008 (−0.112–0.097)	0.88
		2	0.217 (−0.123–0.557)	0.195
		3	0.036 (−0.142–0.213)	0.688

*Model 1 is crude model without any confounders*.

*Model 2 was adjusted for age, sex, BMI, SBP, DBP, heart ratio, smoking habit, alcohol drinking habit, hypertension, diabetes status, dyslipidemia, medicine, and family history of CAD*.

*Model 3 was adjusted for age, sex, BMI, SBP, DBP, heart ratio, smoking habit, alcohol drinking habit, hypertension, diabetes status, dyslipidemia, medicine, family history of CAD, and laboratory tests including HbA1c, FPG, ALT, AST, UREA, UA, estimated glomerular filtration rate (eGFR), HCY, and lipid profiles*.

*The bold values indicate significant results with P < 0.05*.

Complement C1q activity exhibited a positive correlation with Gensini score in the obstructive CAD group without adjustment for the cofounders (β = 0.083, 95% CI 0.035–0.131, *P* < 0.001; Table 3). After calibrating age, sex, BMI, SBP, DBP, HR, smoking habit, alcohol drinking habit, hypertension, diabetes status, dyslipidemia, medicine, and family history of CAD (model 2), the serum complement C1q activity remained a positive correlation with Gensini score in the obstructive CAD group (β = 0.048, 95% CI 0.011–0.085, *P* = 0.012; Table 3). Complement C1q activity showed no place in the regression model of Gensini score after additional adjustment for laboratory tests (*P* > 0.05; Table 3).

### Logistic Regression Analysis of the Risk of C1q Activity for CAD and 1-Year Restenosis

In the whole cohort of 956 patients, each 1-SD increase (42.8 KU/L) in C1q activity was associated with a 1.322-fold (95 confidence interval [95% CI] (1.168–1.496), Table 4) increased Odds Ratio (OR) of CAD in the crude model. When further adjusted for age, sex, BMI, SBP, DBP, HR, smoking habit, alcohol drinking habit, hypertension, diabetes status, dyslipidemia, medicine, and family history of CAD (model 2), the OR for CAD was strengthened to 1.327 (95% CI 1.067–1.651) per 1-SD increase in C1q activity. Ordinal logistic regression showed that each 1-SD increase in C1q activity associated with 43.6% higher odds of having CAD after adjustment for laboratory tests in model 3. When analyzed with the bottom quartile of C1q as the reference, the OR for CAD was 1.419 in the second quartile, 0.922 in the third quartile, and 1.328 in the top quartile (Table 4).

**Table 4:** Associations of C1q and CAD.

**Models**	**CAD**	**Control (*N*)**	**OR (95% CI)**	***P***
Each 1KU/L C1q increase	956	677	1.007 (1.004–1.01)	**<0.001**
Each 1-SD (42.8KU/L) increase	956	677		
Model 1	956	677	1.322 (1.168–1.496)	**<0.001**
Model 2	956	677	1.327 (1.067–1.651)	**0.011**
Model 3	956	677	1.436 (1.052–3.731)	**0.032**
Quartiles				
Model 1				
Q1 (<160.5)	170 (25.1)	247 (25.8)	1 (Reference)	1 (Reference)
Q2 (160.5–186.8)	205 (30.3)	193 (20.2)	1.419 (1.12–1.798)	**0.004**
Q3 (186.8–275.1)	174 (25.7)	235 (24.6)	0.922 (0.726–1.17)	0.504
Q4 (>275.1)	128 (18.9)	281 (29.4)	1.328 (1.047–1.684)	**0.02**
Model 2				
Q1 (<160.5)	170 (25.1)	247 (25.8)		
Q2 (160.5–186.8)	205 (30.3)	193 (20.2)	1.404 (1.08–1.768)	**0.002**
Q3 (186.8–275.1)	174 (25.7)	235 (24.6)	0.931 (0.712–1.15)	0.5
Q4 (>275.1)	128 (18.9)	281 (29.4)	1.325 (1.041–1.680)	**0.01**

*Model 1 is crude model without any confounders*.

*Model 2 was adjusted for age, sex, BMI, SBP, DBP, heart ratio, smoking habit, alcohol drinking habit, hypertension, diabetes status, dyslipidemia, medicine, and family history of CAD*.

*Model 3 was adjusted for age, sex, BMI, SBP, DBP, heart ratio, smoking habit, alcohol drinking habit, hypertension, diabetes status, dyslipidemia, medicine, family history of CAD, and laboratory tests including HbA1c, FPG, ALT, AST, UREA, UA, eGFR, HCY, and lipid profiles*.

*The bold values indicate significant results with P < 0.05*.

In the cohort 485 patients underwent PCI treatment and were followed by 12 months to track the restenosis ratio. The restenosis ratio was 35.7% (173/485). In the crude model, the three ordinal levels of C1q were estimated as risk factors for 1-year restenosis after PCI (OR = 3.11, 95% CI 1.214–7.969, OR = 3.809, 95% CI 1.467–9.887, and OR = 3.4, 95% CI 1.368–8.451 *P* < 0.05, Table 5). After adjustment for age, sex, BMI, SBP, DBP, HR, smoking habit, alcohol drinking habit, hypertension, diabetes status, dyslipidemia, medicine, and family history of CAD (model 2), the ORs were attenuated to 3.272 the top quartile levels of C1q, and increased to 3.809 in the third quartile levels of C1q (*P* < 0.05, Table 5). After additional adjustment for laboratory tests (model 3), the association remained statistic significant (OR = 3.544, 95% CI 1.089–12.702, and OR = 2.837, 95% CI 1.049–9.477. *P* < 0.05, Table 5).

**Table 5:** Associations of C1q and 1-year re-stenosis after coronary revascularization.

**Models**	**re-stenosis ratio *n* (%)**	**OR (95% CI)**	***P***
Each 1KU/L C1q increase	173/485 (35.7)	0.996 (0.992–1)	**0.044**
Quartiles			
Model 1			
Q1 (<160.5)	45/127 (35.4)	1 (Reference)	1 (Reference)
Q2 (160.5–186.8)	41/102 (40.2)	3.11 (1.214–7.969)	***0.018***
Q3 (186.8–275.1)	81/216 (37.5)	3.809 (1.467–9.887)	***0.006***
Q4 (>275.1)	6/40 (15)	3.4 (1.368–8.451)	***0.008***
Model 2			
Q1 (<160.5)	45/127 (35.4)	1 (Reference)	1 (Reference)
Q2 (160.5–186.8)	41/102 (40.2)	2.646 (0.999–7.013)	0.050
Q3 (186.8–275.1)	81/216 (37.5)	4.221 (1.563–11.398)	***0.004***
Q4 (>275.1)	6/40 (15)	3.272 (1.276–8.391)	***0.014***
Model 3			
Q1 (<160.5)	45/127 (35.4)	1 (Reference)	1 (Reference)
Q2 (160.5–186.8)	41/102 (40.2)	1.759 (0.492–6.293)	0.385
Q3 (186.8–275.1)	81/216 (37.5)	3.544 (1.089–12.702)	***0.048***
Q4 (>275.1)	6/40 (15)	2.837 (1.049–9.477)	***0.046***

*Model 1 is crude model without any confounders*.

*Model 2 was adjusted for age, sex, BMI, SBP, DBP, heart ratio, smoking habit, alcohol drinking habit, hypertension, diabetes status, dyslipidemia, medicine, and family history of CAD*.

*Model 3 was adjusted for age, sex, BMI, SBP, DBP, heart ratio, smoking habit, alcohol drinking habit, hypertension, diabetes status, dyslipidemia, medicine, family history of CAD, and laboratory tests including HbA1c, FPG, ALT, AST, UREA, UA, eGFR, HCY, and lipid profiles*.

*The bold values indicate significant results with P < 0.05*.

### ROC Curve

**Figure 2:**
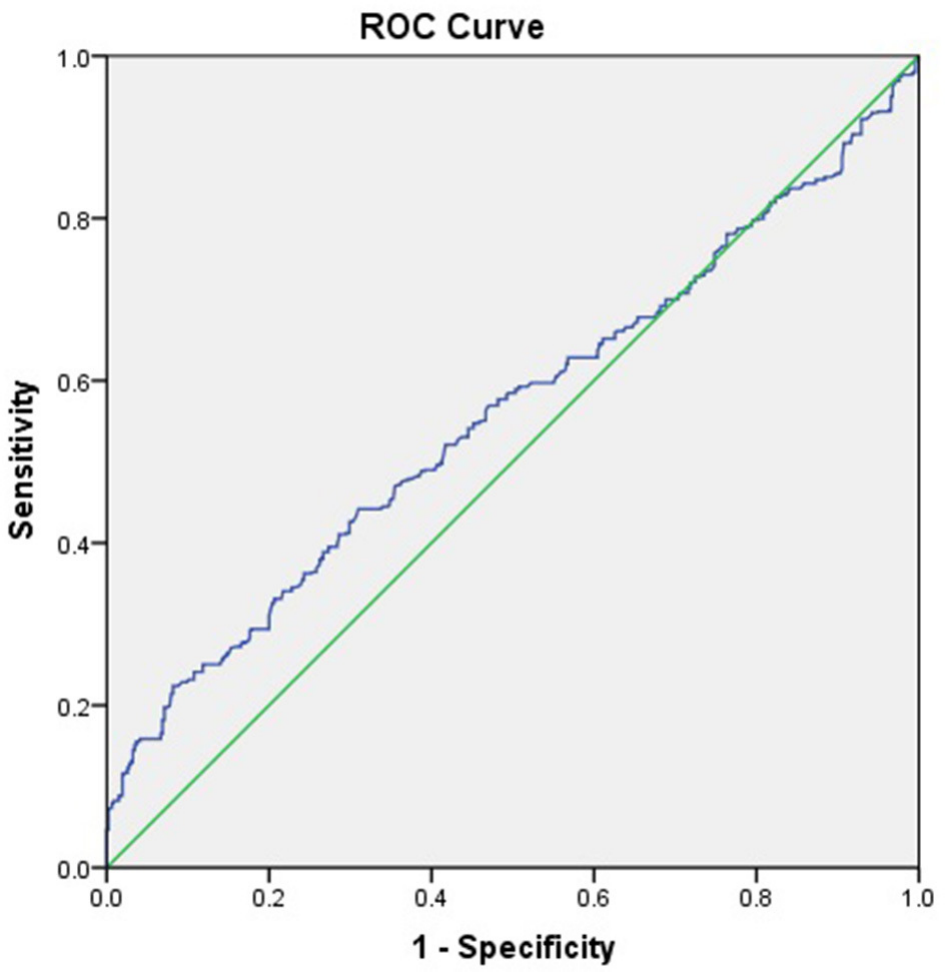
ROC curve of CAD by serum complement C1q activity.

The ROC curve of complement C1q activity as a predictor of CAD suggested that the C1q had low value in predicting the incidence of CAD (C1q = 197.5 KU/L, AUC = 0.558, 95%CI = 0.525–0.592, *P* = 0.001, sensitivity 44.2 %, Specificity 69.0%; Figure 2).

## Discussion

Ischemic heart disease (IHD) represented a large burden on individuals and health care resources worldwide (22). Traditionally, it was equated to coronary artery disease (CAD). However, clinical, angiographic, and autoptic found just some cases were caused by severe or complicated atherosclerotic plaques (23). A large percentage of patients with IHD had minimal or no epicardial coronary vascular disease. In our study, we also found that there was a higher ratio of stable angina in the CAD group without severe or complicated atherosclerotic plaques. IHD hides a multifaceted and complex pathophysiological paradigm including micro and macrovascular dysfunction, atherosclerotic plaque rupture, inflammation, endothelium dependent and independent dysfunction, ion channels (24), and nervous system impairment (25, 26).

Complement C1q was an initiator of the classic pathway (2). However, C1q plays an important role independent of complement activation in many systems. C1q plays a dual role in the chronic inflammatory disease of atherosclerosis. On the one hand, C1q helps maintain the size and complexity of early atherosclerotic lesions by docking on phagocytes and clearing apoptotic cells (27). Apoptosis is associated with the development of human atherosclerosis lesion necrotic core as well as instability of complex plaques. On the other, complement activation via C1q exacerbates pathology in the atherosclerotic lesion in later stages of the disease (28, 29). Upregulating inflammatory signaling in endothelial cells and leucocytes contributes to the development and rupture of vulnerable plaque as well as consequent acute coronary syndrome (30). C1 inhibitor limited neointimal plaque formation and inflammation. This might involve blockade of complement activation, inhibition of leukocyte recruitment, and reduced triglyceride levels, thus providing a multimodal approach to treat arterial disease (31–33).

In this work, we found complement C1q activity in obstructive CAD and non-obstructive CAD groups was significantly higher than the control group. This result was inconsistent with part of previous reports. Xiao-ning Ni reported that level of complement C1q in AMI group was lower significantly than control group and unstable angina group (13). Cavusoglu found that the reduced baseline plasma levels of complement C1q were strong predictors of all-cause mortality in a population of either known or suspected CAD and emerged as a strong and independent predictor of all-cause mortality at 10 years (34). There are several reasons for the inconsistency. First, previous studies only enrolled high-risk participants who were referred for coronary angiography, which may cause selection bias that influences the stability of results. Second, the CAD groups were categorized according to different standards. Xiao-ning Ni used the patients with coronary artery stenosis <50% showed by CAG as control group. Third, the previous studies measured the C1q quantity, and in our study we tested the C1q activity. In addition, the sample size was relatively small in these two previous studies.

Consisted with the previous study (13), we confirmed that complement C1q activity was not correlated with Gensini score in the obstructive CAD group after adjustment for confounders. Complement C1q activity showed no place in the regression model of Gensini score after additional adjustment for laboratory tests. Greater C1q activity was significantly correlated with higher TC levels (β = 0.001, *P* = 0.009) and TG levels (β = 0.002, *P* = 0.031). This correlation may explain part of the disappeared association between C1q activity and Gensini score in the model 3 with adjustment for laboratory tests including lipid profiles.

In order to further explore the association of C1q activity and coronary stenosis in patients with obstructive CAD, this study constructed a logistic regression model and found that C1q activity was associated with an increased OR of obstructive CAD and 1-year restenosis after revasculation. Complement deposition and activation might in fact be the first steps in lesion initiation in the arterial wall, even preceding monocyte infiltration (35, 36). C1q has been confirmed existed in atherosclerotic lesions. C1q is expressed by dendritic cells, macrophages, foam cells, and endothelium cells in atherosclerotic arterial wall (35, 36). The production and deposition of C1q activated the complement system, promoting the formation of membrane attack complex C5b-9, inducing the activation and proliferation of smooth muscle cells and endothelial cells, and promoting the continuous formation of plaques through positive feedback and aggravating vascular stenosis (37). Complement C1q-induced arterial remodeling and arteriosclerosis in patients with hypertension by activating β-catenin signaling (38). The pro-atherosclerotic effect of C1q might explain that the higher activity of C1q was related to the onset of obstructive CAD.

C1q activity in non-obstructive CAD groups was also significantly higher than the control group. Non-obstructive CAD groups represented patients with coronary microvascular disease (CMD). CMD refers to pathologic changes within the small vessels of the coronary circulation, in the absence of obstructive lesions in the major vessels. Contemporary evidence strongly supported the coexistence of CMD with atherosclerosis in some patients (39–41). The impaired endothelial function is a hallmark of IHD (42). Endothelial dysfunction is present in CMD, and this can lead to complement activation. Blood contact with a damaged endothelium would lead to a certain degree of complement system activation (43). Platelets might have an ability to interact with both the classical and alternative pathways of complement activation (44). Higher level of terminal complement complex (TCC) C5b-9 was found in plasma of CMD group compared to those with angiographically proven coronary atherosclerosis (45). However, no parallel activation of the classical or the alternative complement pathway was observed in the Horvath study. Low levels of several lectin pathway products might reflect upstream consumption and consequent, downstream terminal complement complex activation. Complement activation might contribute to the increased cardiovascular risk of non-obstructive CAD by promoting endothelial and microvascular dysfunction.

Stenting is one of the most common procedures used to treat stenosis. Compared with conservative treatment, invasive treatment reduced mortality after acute myocardial infarction (46). However, the full hemocompatibility of the different kinds of stents was not yet reached (47, 48). The constant insult of vessel wall injury due to the metal stent resulted in the non-specific inflammatory response, inducing the migration of smooth muscle cells and myofibroblasts to tunica intima (49–51). PCI may induce a local inflammatory response that contributes to restenosis. The damaged intima integrity, causing exposure of sub-cellular components to the extracellular milieu, attracts C1q, leading to activation of the downstream effectors of complement C1q (52), which has been shown to play a role in enhancing the phagocytosis of immune complexes and apoptotic cells (53, 54). In this regard, PCI might enhance the pro-atherosclerotic effect of C1q activity by inducing non-specific inflammatory response. The association between baseline C1q activity and 1-year restenosis remained statistic significant after adjustment for age, sex, BMI, SBP, DBP, HR, smoking habit, alcohol drinking habit, hypertension, diabetes status, dyslipidemia, medicine, and family history of CAD and laboratory tests (Regression analysis model 2 and 3). Although our data could not elucidate the causality between the C1q activity and pathogenesis of 1-year restenosis, the statistic significant association suggested that higher C1q activity at baseline might predict an increased OR of 1-year restenosis after revasculation.

On the other hand, the increase of C1q activity may be a kind of immune regulation. The increased C1q activity inhibited local inflammatory response in peripheral blood by combining with adiponectin. Therefore, it is an active defense against acute cardiovascular events and helps maintain autoimmune tolerance (55). Regrettably, ROC curve suggested that the accuracy in CAD by serum complement C1q activity was low.

In conclusion, complement C1q activity in obstructive CAD and non-obstructive CAD groups was significantly higher than the control group. Greater C1q activity was significantly correlated with higher TC and TG levels. Complement C1q activity exhibited a positive correlation with Gensini score in the obstructive CAD group, especially in the obstructive CAD with two stenosis vessels. C1q activity was associated with an increased OR of CAD and 1-year restenosis after revasculation. However, complement C1q activity was not correlated with Gensini score in the obstructive CAD group after adjustment for confounders. C1q activity has low value in predicting the incidence of CAD.

Our study has limitations. First, although we used a large sample size, we could not establish a causal relationship between C1q activity and obstructive CAD owing to our observational design. Second, the observation of the significant association between C1q and higher TC and TG levels should be interpreted cautiously, because all cardiovascular biomarkers were measured at a single time point, which might bias the results. Third, 1 year of follow-up was not long enough to assess the association of C1q activity with long-term outcomes after coronary revascularization. Finally, the mechanism of the interaction between complement C1q activity and atherosclerosis in patients with coronary heart disease is not clear.

## Data Availability Statement

The original contributions generated for this study are included in the article/Supplementary Material, further inquiries can be directed to the corresponding author/s.

## Ethics Statement

The studies involving human participants were reviewed and approved by The Zhengzhou University Committee. The patients/participants provided their written informed consent to participate in this study.

## Author Contributions

SG performed the experiments, analyzed the data, prepared figures and tables, authored or reviewed drafts of the paper. XM performed the data collection and analysis. XL, HO, and YG performed the laboratory tests and clinical data collection. LM prepared figures and tables, reviewed drafts of the paper, and approved the final draft. All authors contributed to the article and approved the submitted version.

## Conflict of Interest

The authors declare that the research was conducted in the absence of any commercial or financial relationships that could be construed as a potential conflict of interest.
