# Supplementary material for: Serum Complement C1q Activity Is Associated With Obstructive Coronary Artery Disease
Source: Front Cardiovasc Med. 2021 Apr 29;8:618173. doi: 10.3389/fcvm.2021.618173 (PMC8116493; doi:10.3389/fcvm.2021.618173)
Supplement: Supplementary file 1 [file Table_1.docx]

| Supplementary Table 1 Clinical characteristics of patients and controls | | | | |
| --- | --- | --- | --- | --- |
|  | non-obstructive CAD(n=153) | Obstructive CAD (n=803 ) | Control (n=677 ) | P |
| Smoking n(%) | 43 (28.1) | 279 (34.7) | 149 (22) | <0.001 |
| Drinking n(%) | 46 (30.1) | 242 (30.1) | 136 (20.1) | <0.001 |
| male n(%) | 86(56.2) | 478 (59.5) | 402 (59.4) | 0.074 |
| age（years） | 60.27±11.56 | 60.54±10.29 | 59.09±11.52 | 0.81 |
| BMI(kg/m2) | 23.86±2.4 | 23.96±2.33 | 22.93±2.12 | <0.001 |
| SBP(mmHg) | 129.79±19.71 | 128.82±17.38 | 121.26±9.63 | <0.001 |
| DBP(mmHg) | 78.54±13.28 | 77.72±10.93 | 75.23±8.91 | <0.001 |
| HR（heart ratio） | 75.44±10.75 | 75.12±10.19 | 70.55±5.61 | <0.001 |
| HbA1C(%) | 5.98±1.12 | 6.19±1.31 | 5.44±0.48 | <0.001 |
| FPG (mmol/L) | 5.44±1.76 | 5.57±1.89 | 5.1±0.77 | <0.001 |
| ALT(U/L) | 30.25±33.92 | 43.25±189.45 | 21.18±12.87 | <0.001 |
| AST(U/L) | 29.13±27.18 | 44.62±302.62 | 22.16±8.96 | 0.007 |
| TBIL(µmol/L) | 14.4±22.31 | 10.81±7.12 | 11.51±5.17 | 0.001 |
| BILD (µmol/L) | 7.11±18.39 | 4.78±3.58 | 5.11±1.89 | 0.001 |
| GGT(U/L) | 41.17±53.48 | 33.61±34.15 | 29.37±43.26 | 0.01 |
| TP(g/L) | 66.94±6.29 | 65.64±5.37 | 73.54±3.89 | <0.001 |
| ALB(g/L) | 41.42±4.48 | 40.66±4.14 | 47.07±2.79 | <0.001 |
| ALP(U/L) | 77.64±51.32 | 75.6±24.88 | 70.11±19.66 | 0.002 |
| TC (mmol/L) | 4.04±1.03 | 3.81±1.04 | 4.62±0.84 | <0.001 |
| TG (mmol/L) | 1.84±1.65 | 1.6±1.14 | 1.4±0.83 | <0.001 |
| HDL (mmol/L) | 1.1±0.34 | 1.03±0.29 | 1.31±0.3 | 0.027 |
| LDL (mmol/L) | 2.33±0.93 | 2.14±0.85 | 2.79±0.75 | <0.001 |
| APOA (g/L) | 1.21±0.46 | 1.15±0.26 | 1.44±0.25 | 0.067 |
| APOB (g/L) | 0.92±0.52 | 0.93±1.92 | 0.82±0.2 | 0.477 |
| Lpa (g/L) | 0.22±0.25 | 0.31±1.11 | 0.19±0.19 | 0.07 |
| HCY (umol/L) | 14.5±6.8 | 14.98±8.68 | 13.72±8.52 | 0.04 |
| hsCRP (mg/L) | 3.91±9.14 | 7.55±21.16 | 1.34±1.32 | 0.03 |
| CREA (μmol/L) | 72.26±23.9 | 80.7±67.75 | 73.28±14.98 | 0.012 |
| Urea (mmol/L) | 5.85±3.01 | 5.83±2.96 | 4.93±1.29 | <0.001 |
| UA (mmol/L) | 309.47±99.24 | 304.17±105.06 | 320.76±85.65 | 0.014 |
| eGFR | 91.06±19.02 | 88.7±19.72 | 96.03±14.36 | <0.001 |
| C1q (kU/L) | 195.42±51.25 | 195.52±48.31 | 183.44±31.75 | <0.001 |
| IRON (µmol/L) | 13.13±5.69 | 13.4±6.4 | 20.93±8.15 | <0.001 |
| FERR (ng/mL) | 380.42±452.29 | 328.34±352.28 | 196.16±174.13 | <0.001 |
| UIBCI (µmol/L) | 32.73±7.05 | 33.97±7.51 | 37.52±12.12 | <0.001 |
